# Current Genetic Epidemiology of *β*-Thalassemias and Structural Hemoglobin Variants in the Lazio Region (Central Italy) Following Recent Migration Movements

**DOI:** 10.1155/2010/317542

**Published:** 2010-10-05

**Authors:** Antonio Amato, Maria Pia Cappabianca, Alessia Colosimo, Maria Perri, Paola Grisanti, Ivo Zaghis, Donatella Ponzini, Maria Lerone

**Affiliations:** ^1^ANMI Onlus, Centro Studi Microcitemie, Rome, Italy; ^2^Department of Comparative Biomedical Sciencies, University of Teramo, Italy

## Abstract

The aim of this study was to describe the changing pattern of mutational spectrum of *β*-thalassemia (*β*-thal) in the Lazio region (Central Italy), as consequence of recent demographic variations. From 1994 until present, 256 immigrant subjects with hemoglobin disorders (including 191 heterozygotes and 65 homozygotes or compound heterozygotes) coming from 44 different foreign countries, have been molecularly characterized. 14 *β*-globin gene mutations were identified and their frequencies reflect different ethnic origins: 8 of these mutations account for 76.97% of all molecular defects, while 6 of them are much rare, representing less than 2% of the total. These data differ, both in type and percentage, from the mutational spectrum detected in the native population in 1995. Since a few defects are prevalent in each country, a proper strategy for the identification of mutations in immigrant individuals relies on the prior knowledge of their frequency in native ethnic group.

## 1. Introduction

The inherited disorders of hemoglobin (including structural hemoglobin variants and *α*- and *β*-thalassemias) show a marked molecular variability in the different ethnic groups that mainly results from heterogeneous mutations occurring at the *α*- and *β*-globin loci. At present, more than 600 and 700 mutations involving the *α*-globin and the *β*-globin genes, respectively, have been described worldwide [1].

In the Lazio region (Latium) of Central Italy, *β*-thalassemia (*β*-thal) is relatively common among the native population with a prevalence of 2.4% [2]. The “Centro Studi Microcitemie Roma” (CSMR) has been leading since 35 years a prevention program of hemoglobinopathies and thalassemias in Latium by a strategic plan including three different types of actions: information to the population, healthy carriers detection, and genetic counselling for at-risk couples [3]. 

The epidemiology of hemoglobin disorders has been drastically changed since the early 1980s–1990s [4] when a consistent and progressive migration from emerging countries has started in Italy, particularly in Latium. For this reason, it is fundamental to adopt novel strategies to include immigrant people in each regional prevention program. To this aim, to correctly evaluate the current mutational spectrum of *β*-thal and structural hemoglobin variants in Latium, a cohort of 256 immigrant subjects with hemoglobin disorders, who are now resident in the region, has been molecularly characterized. These subjects have been analyzed by molecular studies, because they were carriers, partners of couples at risk of having children affected with Thalassemia major or intermedia, or because they were affected.

 In addition, a comparison with a previous study that analyzed 203 prospective at-risk couples, who were native of Latium [5], has been carried out. This paper allows of verifying the changes occurred at the molecular level in *β*-thal diagnostics in this region, and it is aimed at better directing the proper strategy, adopting, if necessary, new types of methodologies.

## 2. Materials and Methods

5722 individuals (including affected patients, *β*-thal and hemoglobinopathies carriers, and their partners with the aim of detecting silent forms of thalassemia) have been molecularly analyzed for the *β*-globin genes. Of these subjects, 256 were immigrant who resulted *β*-thal carriers or affected individuals after hematological examination, carried out by cell counter Advia 2120 Hematology Systems (Bayer Health Care-Diagnostics Division-Milan, Italy) and hemoglobin fractions study, carried out by Variant II (Bio-Rad, Laboratories, Hercules, CA, USA). The study was performed after obtaining patients' consent, all register office data, and geographical provenience of the family. 

Genomic DNA was extracted from peripheral EDTA anticoagulated whole blood by salting out [6]. The molecular analysis of the *β*-globin gene was carried out by several PCR-based techniques (ARMS-PCR, gap-PCR, restriction endonuclease analysis and direct DNA sequencing) according to the defect to investigate. Since the most common *β*-thal causative defects are point mutations [1], we prevalently used the Amplification Refractory Mutation System (ARMS) [7]. To perform this analysis, allele-specific primers, useful to selectively amplify the most common mutations that are prevalent in the countries of origin of all patients, were designed. Rare mutations were detected by genomic direct sequencing (Beckman Coulter CEQ TM 8000 Genetic Analysis System), using primers covering the full genomic region of the *β*-globin gene [8].

## 3. Results

Between 1994 and 2007, at the CSMR, a total of 167,235 individuals has been examined by haematological, haemoglobin, and biochemical analyses. Of this total number, 10, 353 were immigrant, indicating a progressive increase in the above- mentioned years (from 2.7% until 9.8%). Among the 10,353 individuals, 7869 resulted to be wild type while 2484 were either carriers or affected [9] (Table 3(a)).

The 256 individuals, molecularly examined, were mainly coming from East Europe (102 subjects: Albania was included in this group for geographical reasons) and Africa (86 subjects), and in lower amount from Asia (60 subjects) and Central-Southern America (8 subjects). These 256 individuals were divided in 110 healthy *β*-thal carriers, 81 carriers of hemoglobin variants, and 65 homozygous or compound heterozygous patients.

From familiar data involving 191 healthy carriers analyzed at our molecular biology laboratories from 1994 until present (110 healthy *β*-thal carriers and 81 carriers of several hemoglobinopathies), 40.31% were coming from East Europe, mainly from Albania; 30.37% from Africa, mainly from Egypt and Central-Western Africa (Senegal, Cote d'Ivoire, Ghana, Nigeria, Congo, Burkina Faso); 25.65% from Asia (Asian and Middle Eastern countries), mainly from Bangladesh (Table 1).

This novel heterogeneous population of immigrants has deeply modified the mutational spectrum of *β*-thal alleles that was observed in this Italian region 14 years ago. In fact, a previously reported study, carried out on 406 individuals of Latium descendant, detected 8 more common mutations (Cd 39, IVSI-110, IVSII-745, IVSI-6, IVSI-1, IVSII-1, Frameshift 6, and Hb Lepore) that accounted for 93.4% of all *β*-thal alleles in the region [5]. Conversely, the present paper shows that 8 partially different mutations (HbS, IVSI-110, HbE, cod 39, IVSI-5, IVSI-6, HbC, and IVSI-1) account for 76.97% of all *β*-globin molecular defects in the foreign population (Table 1). Due to the wide ethnic heterogeneity of these heterozygous subjects, other molecular defects were detected. These include several abnormal hemoglobins (that are usually rare), some deletional mutations (Sicilian, Indian, Filipino, and a novel 12-bp deletion at the beginning of the *β* globin second intron found in a woman of Indian descent (10)) and other rare *β* thal alleles (Table 1). 

A few of the most common mutations in Latium are also present in patients originating from East Europe [10], although with different frequencies. In addition, some particular hemoglobin variants such as HbS, that in Italy are predominant only in Southern populations [11], were also detected in 4 subjects accounting for 5.19% of the total (Table 1). 

In Asian and Middle-Eastern populations, the HbE is mainly present [12] (17 subjects out of 49, corresponding to 34.69%) and the IVSI-5 (G→C) mutation [13] (16 subjects corresponding to 32.65%), that in Latium is present only in the G→A variant with a frequency of 0.45% [5].

In African populations, the hemoglobin variants are the most common alterations [14], including 36 subjects with HbS (62.07%), 6 subjects with HbC (10.34%), and 3 subjects with Hb Knossos [15] (5.17%) (Table 1).

All the mutations that have been detected in 191 healthy carriers were also revealed in different combinations in 65 relatives, who were homozygous or compound heterozygous patients (Table 2).

## 4. Discussion

Latium is the Italian region with the highest incidence of most recent immigration. From published data in 2009 by Caritas/Osservatorio Romano sulle Migrazioni [16], out of a total of 11.69% immigrants who are resident in Latium [17], 30.70% are coming from Romania, 6.81% from Philippines, 5.39% from Poland, 4.97% from Albania, 3.33% from Ukraine, and 3.05% from Peru. These data do not perfectly match with the ethnic variety of individuals who arrived at our CSMR laboratory, for cultural, social, and also genetic reasons. In fact, the Philippine population is absent in our cohort of samples because in that country is prevalent the *α*-thal and not the *β*-thal [18], while Romanians are less represented for the rare presence (0.5%) of *β*-thal in that country [19].

During the last 15 years, the number of molecular analyses carried out at CSMR on foreign patients or healthy carriers has been progressively increased; in a five-year period (2004–2008) has been quintupled, if compared to the first five years (1994–1998) (Table 3(b)). Although a few and very comprehensive studies have determined the influence of recent immigration movements by updating the spectrum of *β*-globin mutations in Northern and Western Europe, or in the United Kingdom [20, 21], such a similar epidemiological study regarding the changing spectrum of mutations in Italy has not been carried out. This paper is aimed at fulfilling this lack of information by determining the novel spectrum of *β*-globin mutations observed in immigrant populations in the Latium region from 1994 until present. In our Molecular Biology Laboratory, from 1994 to present, we have detected 256 foreign subjects with hereditary hemoglobin disorders (4.47% of the total molecular analysis carried out in the considered 15 years) (Table 3(b)). If compared to the molecular analyses carried out in 1995 and involving only heterozygotes who were native of Latium [5], the present research highlighted a wider heterogeneity of all mutations in immigrant subjects. Although also in this cohort of individuals some mutations are more common, it is evident the presence of structural hemoglobin variants that were absent in the Latium population in the recent past. As expected, a similar heterogeneity was also evident in carriers of two molecular alterations (i.e., patients affected with thalassemia major, thalassemia intermedia, and hemoglobinopathies) (Table 2).

The novel mutations that in the recent past were never described in the Latium native population are obviously common in the countries of origin of the immigrant *β*-thal carriers; for example: cod 82-83 (-G) in Albania, cod 10 (C→A)/cod 16 (-C) in Afghanistan, cod 36-37 (-T) in Santo Domingo, cod 41-42 (-TCTT) in Bangladesh, cod 51 (-C) in Romania, −29 (A→G) in Venezuela, and IVS II-654 (C→T) in China. Regarding the hemoglobin variants, that are considered of scarce clinical relevance in our region, with the exception of the Hb Lepore (2.05%), the HbS is more frequent in African descendant individuals, the HbE is common in Asian subjects, while other variants such as HbC, Hb Lepore, and Hb Knossos are evident in individuals originating from countries in which these mutations are common.

Finally, some variants that are not relevant from a clinical point of view including Hb Kenitra in a subject from Nigeria, Hb G-Coushatta in a Iranian subject, Hb Hoshida in an Hungarian subject, have been detected by direct sequencing of the *β*-globin gene. 

 Of course, all the detected molecular defects only partially reflect the real variety that is present in each native country [22].

Nowadays, a suitable strategy for identifying the *β*-globin mutations needs the knowledge of the mutations, common or rare, that are present in the ethnic group to which the individuals under examination belong to. This information simplifies the diagnostic strategy because it allows to design the appropriate allele-specific primers, the digestion restriction enzymes, or the appropriate probes to detect the mutations that are more common in the patient's country of origin. In addition, it is always fundamental to perform family studies for the identification of heterozygous mutations, in the case of patients carrying the association of two *β*-globin defects, and also for understanding the segregation of different genes that may modify the phenotype. The mutations that remain uncharacterized may be detected by direct automatic sequencing analysis of genomic DNA. It is becoming evident that in the present condition of uninterrupted and constant migration of foreign individuals, who are carriers of mutations that are rare in the Italian indigenous population, the use of commercial kits is not sufficient for the mutation detection in a such heterogeneous cohort of subjects. 

In the Latium region of its competence, and starting from 1994, the CSMR was able to reduce to zero the number of affected offspring from unaware native couples, using a protocol that included a mass screening of boys and girls (of about 13 years old) who were attending the secondary school. This screening, joined to an adequate sanitary information, has allowed the precocious identification of healthy carriers. In addition, postdiagnosis genetic counselling has been able to give to the couples at risk all the medical information and the alternative options of prevention. In this way, all the informed carriers may decide by themselves according to their ethical or religious opinions.

Today, the main objective of CSMR is to inform also the foreign population of the risk [22, 23] and the possibility of prevention [24] to provide means of hematological and molecular diagnosis and to offer a proper service of genetic counselling.

## Figures and Tables

**Table 1:** Frequency distribution of *β* alleles (*β* thal and Hb variants) in 191 heterozygous immigrants.

Molecular defects	Frequency (*n*:191)		Europe (*n*:77) 40.31%	Africa (*n*:58) 30.37%	Asian countries (*n*:42) 21.99%	Middle Eastern countries (*n*:7) 3.66%	South Central America (*n*:7) 3.66
	*n*	%					
Hb S	41	21.47	4	36			1
IVS-I-110 (G→A)	30	15.71	27	3			
Hb E	17	8.90			17		
Cod 39 (CAG→TAG)	17	8.90	17				
IVS-I-5 (G→A, G→C)	16	8.38			14	2	
IVS-I-6 (T→C)	11	5.76	8	1		2	
Hb C	8	4.19		6			2
IVS-I-1 (G→A)	7	3.66	5	1			1

Cod 82-83 (-G)	3	1.57	3				
Hb Knossos	3	1.57		3			
Hb Lepore	3	1.57	2		1		
IVS-II-848(C→A)	3	1.57	1	2			
Cod 41-42(-TTCT)	2	1.05			2		
Cod 51 (-C)	2	1.05	2				
Deletional mutations	8	4.19	1	2	4		1
Other abnormal Hbs	9	4.71	3	2	3	1	
Other rare mutations	11	5.76	4	2	1	2	2

**Table 2:** Interactions between different alleles in the *β* globin genes (65 immigrant patients).

*β* ^0^/*β* ^0^	*β* ^0^/*β* ^+^	*β* ^+^/*β* ^+^−*β* ^++^	Hb variants
Cd 39 homozygous (4)	Cd 39 / IVS-I 110 (2)	IVS-I 110 homozygous (5)	Hb S/Hb S (19)
Cd 39/IVS-I 1 (1)	Cd 8/IVS-I 6 (1)	IVS-I 110/Hb Knossos (1)	Hb S/Hb C (3)
Cd 10/Cd 16 homozygous (1)	Cd 41-42/IVS-I 1 (1)	IVS-I 110/IVS-II 848 (2)	Hb S/Cd 39 (1)
Cd 6 homozygous (1)	IVS-I 1/IVS-I 110 (1)	IVS-I 110/-87 (1)	Hb S/IVS-I 110 (2)
Cd 39/Cd 82-83 (1)	Hb Lepore/IVS-I 5 (1)	IVS-I 110/Hb E (1)	Hb S/Cd 82-83 (1)
Cd 51/Hb Lepore (1)		IVS-I 6/-30 (1)	Hb C homozygous (3)
		Hb E homozygous (5)	
		Hb E/deletion (1)	
		IVS-I 6 homozygous (3)	
		IVS-I 5 homozygous (1)	

**Table:** (a) Laboratory tests for provisional diagnosis (normal, anemic, iron deficient, carrier, and affected individuals)

Years	Examined subjects (*n*)	Immigrants			
		(*n*)	%	Carriers of affected	%
1994–2007	167.235	10.353	6.2	2484	24.0

**Table:** (b) Molecular tests for definitive diagnosis (carriers and affected individuals)

Years	Examined subjects (*n*)	Immigrants	
		(*n*)	**%**
1994–1998	950	26	2.74
1999–2003	2,047	93	4.54
2004–2008	2,725	137	5.03

1994–2008	5,722	256	4.47
